# An Acyl Carrier Protein Gene Affects Fatty Acid Synthesis and Growth of *Hermetia illucens*

**DOI:** 10.3390/insects14030300

**Published:** 2023-03-21

**Authors:** Xiaoyan Peng, Jiawen Liu, Baoling Li, Shengyin Wang, Bosheng Chen, Dayu Zhang

**Affiliations:** The Key Laboratory for Quality Improvement of Agricultural Products of Zhejiang Province, College of Advanced Agriculture, Zhejiang A&F University, Hangzhou 311300, China

**Keywords:** *Hermetia illucens*, acyl carrier protein, fatty acid, growth and development, RNAi

## Abstract

**Simple Summary:**

Fatty acids play essential roles in insect growth and development. The key fatty acid synthesis reaction is catalyzed by fatty acid synthase (FAS). As a cofactor of fatty acid synthesis, however, little is known about the functions of acyl carrier protein (ACP) in insects and how it modulates the composition of fatty acids. We isolated an ACP from the black soldier fly *Hermetia illucens* (Diptera: Stratiomyidae) and investigated the expression patterns as well as functions in fatty acid synthesis and growth of insects. *HiACP* has a classical conserved region of DSLD. Based on the RNAi approach, we found that *HiACP* could regulate the fatty acid synthesis, in particular, reducing the composition of saturated fatty acids. Meanwhile, HiACP could also affect the growth and mortality of *H. illucens.*

**Abstract:**

Acyl carrier protein (*ACP*) is an acyl carrier in fatty acid synthesis and is an important cofactor of fatty acid synthetase. Little is known about ACP in insects and how this protein may modulate the composition and storage of fatty acids. We used an RNAi-assisted strategy to study the potential function of ACP in *Hermetia illucens* (Diptera: Stratiomyidae). We identified a *HiACP* gene with a cDNA length of 501 bp and a classical conserved region of DSLD. This gene was highly expressed in the egg and late larval instars and was most abundant in the midgut and fat bodies of larvae. Injection of *dsACP* significantly inhibited the expression level of *HiACP* and further regulated the fatty acid synthesis in treated *H. illucens* larvae. The composition of saturated fatty acids was reduced, and the percentage of unsaturated fatty acids (*UFAs*) was increased. After interfering with *HiACP*, the cumulative mortality of *H. illucens* increased to 68.00% (*p* < 0.05). *H. illucens* growth was greatly influenced. The development duration increased to 5.5 days, the average final body weights of larvae and pupae were decreased by 44.85 mg and 14.59 mg, respectively, and the average body lengths of larvae and pupae were significantly shortened by 3.09 mm and 3.82 mm, respectively. The adult eclosion rate and the oviposition of adult females were also severely influenced. These results demonstrated that *HiACP* regulates fatty acid content and influences multiple biological processes of *H. illucens*.

## 1. Introduction

Fatty acids play essential roles in animal health and development [1]. They can be esterified by glycerol and serve as energy sources (triglycerides) within adipose tissue. Fatty acids are also present in cell membrane phospholipids involved in cell structure and membrane fluidity. They stimulate signaling processes, including immune response or metabolic functions [2,3]. In insects, fatty acids are crucial for embryonic development, metamorphosis, and reproductive processes [4,5,6]. To obtain fatty acids, insects have evolved a system for their de novo synthesis.

De novo synthesis of fatty acids in insects begins with the transformation of acetyl-CoA to malonyl-ACP [7]. This compound further reacts with acetyl-ACP by a chain-extending reaction. Then, a series of reaction steps, including reduction, dehydration, and desaturation, occurs [8]. Repetition of this reaction cycle results in fatty acids with different carbon chain lengths. This key reaction cycle is catalyzed by fatty acid synthase (FAS), which contains multiple catalytic domains. During the chain-extending reaction, nearly all acyl substrates and intermediates are attached to an acyl carrier protein (ACP) and shuttled to different catalytic sites of FAS [7,9,10,11]. With the help of a phosphopantetheinyl transferase, ACPs can bind to the 4′-phosphopantetheine arm of coenzyme A (CoA) and transfer to the “active form” for delivery of acyl substituents to distinct FAS domains [12,13]. ACP is the central element of FAS and fatty acid metabolism [14]. ACP deficiency in animals can cause visible damage. For example, mutations in ACP-related genes in animal lungs can impair FAS function, reduce the number of long-chain fatty acids, and delay growth [15]. Downregulation of the active form of ACP can impair the lipoylation function and lead to the decreased activity of lipoylated proteins [16]. ACP is also a key player in insect development and lipid metabolism. The mitochondrial ACP gene of *Drosophila melanogaster* is most highly expressed in late embryonic development, and mutations in its ACP isoform can increase mortality [17,18]. However, little is known about the impact of ACPs on the biosynthesis, storage, and composition of the fatty acids of other insect species. Research is needed to evaluate the influence of ACP deficiency on insect growth and development.

*Hermetia illucens* (Diptera: Stratiomyidae) is a saprophagous species widely used for the bioconversion of organic wastes [19,20,21,22,23]. Similar to other edible insects such as mealworms, silkworms, and crickets, *H. illucens* larvae can utilize carbon and nitrate sources in waste and produce high-quality protein, fatty acid, and mineral nutrients [24,25,26]. The fatty acids in *H. illucens* larvae are mainly saturated fatty acids (SFAs), which account for 50% of the total fat [27,28]. Among these, C12:0 has the highest yield, which generally accounts for 40–60% of the crude fat [29]. The SFAs in *H. illucens* larvae are present at a higher level, which is distinctive compared with other insects such as the mealworm (*Tenebrio molitor*) [30]. This composition can be altered by the larval diet [31]. Thus, the *H. illucens* larva is a good source of lauric acid (C12:0), palmitic acid (C16:0), oleic acid (C18:1), linoleic acid (C18:2), and linolenic acid (C18:3) [32,33]. Therefore, uncovering the mechanisms of fatty acid synthesis and storage is required for this beneficial decomposer insect and its associated industry. The conversion efficiency of fatty acids and their composition in *H. illucens* is mainly governed by FAS, highlighting the need to understand the mechanism of this enzyme and its possible use in the waste disposal and recycling industry.

To investigate the impact of FAS on the fatty acid metabolism of *H. illucens*, we focused on the functions of ACP, a key cofactor serving as the intermediate carrier during fatty acid biosynthesis. We developed an RNAi system to investigate the influence of downregulating the *ACP* gene in 3rd instar *H. illucens* larvae. We also used gas chromatography to assess changes in fatty acid composition in *ACP*-inhibited individuals. By determining the effect of the downregulation on larval, pupal, and adult biomass, survival rate, and development duration, we could further estimate the functions of *HiACP* on the survival and growth of *H. illucens* and explore the possibility of enhancing the fatty acid quality of this economic insect species.

## 2. Materials and Methods

### 2.1. Insect Rearing and Sample Collection

*Hermetia illucens* were reared in an artificial incubator. The temperature was set as 28 ± 1 °C; the relative humidity was 70 ± 5%; the light-dark cycle was 8:16 (L:D) [34], and the light intensity was 4800 lx. Wheat bran was used as the main feed, supplemented with fruits and water. Chicken manure was used to stimulate adults for laying eggs. Larvae of six instar (6th instar larvae are also called pre-pupae) [35,36], pupae, adults (male and female, respectively), and eggs were sampled and stored at −80 °C before use. The head, hemolymph, fat body, cuticle layer, and midgut of the 5th instar larvae were thereafter dissected for RNA isolation. RNA was extracted using the Trizol method following the manufacturer’s instructions. Three biological replicates and three technical replicates were prepared for each test [37].

### 2.2. Cloning of HiACP cDNAs

The concentration and A260/A280 value of the extracted RNA were measured by a UV/VIS spectrophotometer (biodrop μ Lite, Cambridge, UK). The integrity of RNA was verified by agarose gel electrophoresis. The cDNA synthesis reaction was carried out by hiscript II 1st strand cDNA synthesis Kit (Vazyme, Nanjing, China). The synthesized cDNA was used as the template for PCR reaction as follows: pre-denaturation at 95 °C for 3 min; denaturation at 95 °C for 15 s; annealing at 60 °C for 15 s; extending at 72 °C for 60 s; extending at 72 °C for 2 min; 35 cycles; holding at 12 °C [38]. Primer sets used for the amplification of *HiACP* are shown in Table 1. The sequences of PCR products were carried out in Tianke High-tech Development Co., Ltd. (Hangzhou, China).

### 2.3. Sequence Analysis and Quantification of the HiACP Gene

The nucleotide sequence of cDNA was transformed into an amino acid sequence by the Translate tool implicated in Expasy. *HiACP* gene sequences of other insect species were downloaded by the NCBI protein database [39]. The physicochemical properties of protein were predicted by Expasy [40]. The signal peptide was predicted using SignalP [41]. Protein transmembrane structure was predicted by TMHMM [42]. Subcellular localization was performed by Euk-multi-2 [43]. Prediction of phosphorylation sites was performed by NetPhos [44]. Protein secondary structure was predicted by Cgi-bin. Multi-sequence alignment was obtained by DNAMAN software [45].

Quantification of the *HiACP* gene was performed by Real-time PCR method. The cDNA was diluted to 100 ng/μL. PCR reactions were prepared by TB GREENTM premix ex taq (Takara). The reaction conditions were set as follows: pre-denaturation at 95 °C for 30 s; 95 °C, 10 s; 60 °C, 30 s, 39 cycles; 95 °C, 15 s; 65 °C, 5 s; 95 °C, 5 s. We used *β-actin* as a housekeeping gene [46]. Table 1 shows the primers used for qRT-PCR. Three biological repeats were prepared in total, and each was repeated three times (technical repeats). The relative transcription level was analyzed using the 2^−ΔΔCt^ method [47].

### 2.4. RNAi Treatment

The DNA of HiACP was amplified from the cDNA of *H. illucens* larvae using the primer set “HiACP” (Table 1). PCR products were purified by the gel extraction kit (Vazyme, Nanjing, China). The concentration of the final DNA product was greater than 60 ng/μL, and the A260/A280 value ranged from 1.8 to 2.0 [48]. The DNA fragments were further used for dsRNA synthesis. Synthesis of dsRNA was performed using T7 high-yield RNA transcription Kit (Vazyme Biotech Co., Ltd.). Primers used in this step were shown in Table 1 (dsACP and dsGFP). For dsRNA injection, the 3rd-instar larvae were starved for 24 h, then washed, dried, and settled on the ice. Before injection, 75% alcohol was used to disinfect the 10 μL microinjector. Subsequently, the tip of the injector was burned by a burner, and the injection was preceded after the needle was cooled down [49]. A total of 1.5 μL of dsRNA was injected into the membrane of the 7–8th segments of the larvae. The treated insects were reared in an incubator for further tests. Daily cumulative mortality (Daily cumulative mortality (%) = cumulative number of dead worms per day/total number of worms × 100%) [50] of RNAi-treated larvae was noted every day. Body weight was measured daily, and larval body length was measured by Leica M165 C stereo microscope. The eclosion rate was calculated as follows: Adult eclosion rate (%) = adult number/total pupae number × 100%. Oviposition rate (%) = the number of eggs/the number of female adults × 100% [51].

### 2.5. Fatty Acid Contents in H. illucens

Larvae were ground to powder with liquid nitrogen. Totally 0.2 g of larvae powder was collected into a glass tube, and 1 μg/mL of C17:0 was added as the internal standard. Total Fatty Acids were extracted by chloroform and dried by N_2_. Methyl esterification was carried out with the methanol-H_2_SO_4_ method; n-hexane was used to extract fatty methyl esters (FAME) [52]. The FAME products were measured by an Agilent 6890N Gas Chromatography gas phase analyzer with a DB-23 column (60 m × 250 m × 0.25 m). The sampling parameter was set as follows: detector temperature, 260 °C; inlet temperature, 270 °C. The heating program was started initially at 100 °C for 13 min, then 10 °C/min to 180 °C for 5 min, 1 °C/min to 200 °C for 20 min, and finally heated at 4 °C/min to 240 °C for 10 min [53]. Helium was used as the carrier gas. The C4–C24 FAME mixture (Supelco 37 F. A. M. E. Mix, Sigma-Aldrich, St. Louis, MO, USA) was used as the standard solution.

### 2.6. Statistical Analysis

All statistical analysis was performed by Prism 9.0. Variations of test groups were calculated by Student’s *t*-test (for two groups) and One-Way ANOVA followed by Tukey’s post-hoc test (pair-wise analysis of multi-groups).

## 3. Results

### 3.1. Sequence Analysis of HiACP

**Figure 1:**
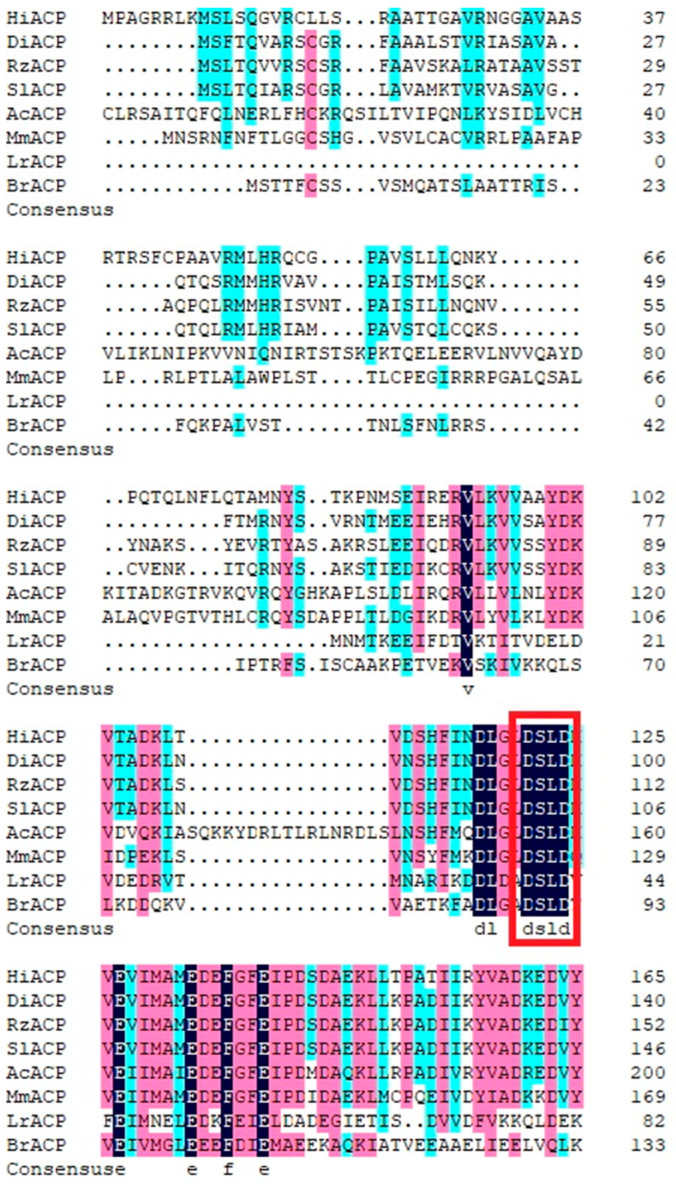
Multiple sequence alignment of *HiACP* with *Drosophila innubila DiACP* (GenBank accession no: XP-034481180.1), *Rhagoletis zephyria RzACP* (GenBank accession no: XP-017477199.1), *Scaptodrosophila lebanonensis SlACP* (GenBank accession no: XP-030385635.1), *Apis cerana cerana AcACP* (GenBank accession no: PBC33684.1), *Mus musculus MmACP* (GenBank accession no: XP-036009503.1), *Lactobacillus reuteri LrACP* (GenBank accession no: CUU12525.1), and *Brassica rapa BrACP* (GenBank accession no: NP-001288921.1). Characters shaded in different colors indicate amino acids with distinct similarity (deep blue, 100%; pink, 75–99%; light blue, 50–74%). The red box indicates strictly conserved motifs in ACPs.

Physicochemical properties and structure analysis of *HiACP* by NCBI [39] showed that the full-length open reading frame of *HiACP* (GenBank accession# OP659030) was 501 bp, and it encoded 165 amino acids. The molecular weight was 18.4 kDa. The negatively charged residue (Asp + Glu) was 20. The positively charged residue (Arg + Lys) was 20. The instability index was 43.38. The fat coefficient was 89.34. The total hydrophobic coefficient was −0.156. A blast of the amino acid sequence of *HiACP* with homologous genes of other species by NCBI showed that it contains a conserved region of DSLD (Figure 1). The cloned *HiACP* genes were blasted by the NCBI database, where species with high similarity ranking and small e-value included *Drosophila innubila* (XP-034481180.1) with 61% similarity and an e-value of 5 × 10^−55^, *Scaptodrosophila lebanonensis* (XP-030385635.1) with 60% similarity and an e-value of 8 × 10^−52^ and *Rhagoletis zephyria* (XP-017477199.1) with 60% similarity and an e-value of 2 × 10^−52^. The physical and chemical properties of the protein were analyzed by ExPASY [40]. TMHMM2.0 [42] predicted that *HiACP* had no transmembrane structure. SignalP 5.0 predicted *HiACP* signal-free peptides. Euk-m PLoc 2.0 [41] predicted that *HiACP* was localized in the mitochondria. SOMPA [43] predicted that *HiACP* had 68.07% alpha helix, 3.61% extended chain, 4.82% beta-turn, and 23.49% irregular curl. NetPhos3.1 Server [44] predicted that *HiACP* had 11 serine phosphorylation sites (10, 12, 20, 37, 41, 59, 81, 87, 112, 122, 143), 10 threonine phosphorylation sites (24, 25, 39, 69, 76, 82, 104, 109, 150, 153), and five tyrosine phosphorylation sites (66, 80, 100, 157, 165).

### 3.2. Expression at Growth Stages and Tissues

**Figure 2:**
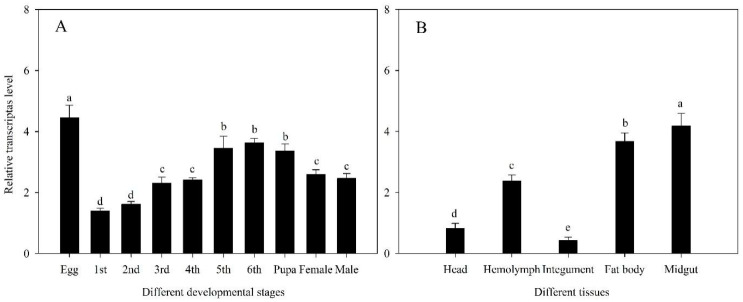
Relative transcript levels of *HiACP* at different developmental stages and in different tissues. (**A**): Relative transcript level of *HiACP* at different developmental stages. Lowercase letters (a–d) represent significant differences (*p* < 0.05) according to Tukey’s multiple range test. (**B**): Relative transcript levels of *HiACP* in different tissues. Lowercase letters (a–e) represent significant differences (*p* < 0.05) using Tukey’s multiple range test. The relative transcript levels of *HiACP* in different developmental stages and different tissues were detected by qRT-PCR (Using *β-actin* as the reference housekeeping gene). The Y-axis values are mean ± SE of relative expression levels. Different lowercase letters on the bars indicate a significant difference between the treatments.

The relative expression levels of *HiACP* in different developmental stages and tissues of *H. illucens* were determined by qRT-PCR. The midgut, integument, hemolymph, fat body, head of 5th instar larvae, and the egg, 1st, 2nd, 3rd, 4th, 5th, and 6th instar larvae, pupae, female, and male adults were tested. The relative expression levels of *HiACP* in the different developmental stages of *H. illucens* were as follows: The relative transcription level in the egg stage was significantly greater than that in other developmental stages (*p* < 0.05). The relative transcript levels of *HiACP* increased as the larval instar increased, but it decreased from the pupal stage to the period after adult eclosion (Figure 2A). The results showed the relative transcript level of *HiACP* in different tissues. The highest expression level was in the midgut (*p* < 0.05), followed by the expression in the fat body (*p* < 0.05) (Figure 2B).

### 3.3. Effects of RNAi of HiACP

**Figure 3:**
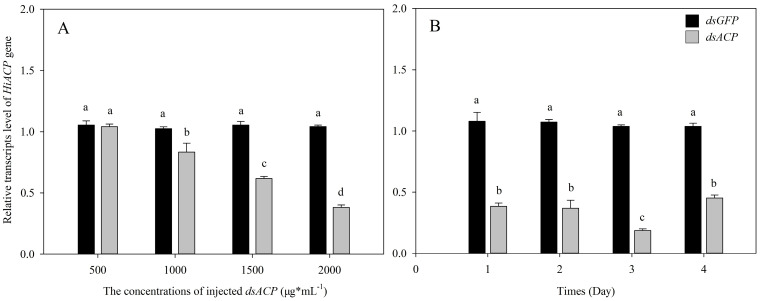
Relative transcript levels of *HiACP* after injection of 1.5 μL *dsACP* in one-day-old 3rd instar larvae with different concentrations and different days. (**A**): Relative transcript levels of *HiACP* 48 h after injection of 1.5 μL *dsACP* in one-day-old 3rd instar larvae with different concentrations. The larvae injected with *dsACP* were collected at 48 h. (**B**): Relative transcript levels of *HiACP* after 1.5 μL (2000 μg·mL^−1^) *dsACP* injection in 3rd instar larvae. The transcript level of *HiACP* was analyzed by qRT-PCR. Mean ± SE was calculated from five biological replicates, and each biological replicate contained ten 3rd instar larvae. Bars marked with different letters indicate significant differences between treatment and control (Student’s *t*-test, *p* < 0.05).

We injected dsRNA into one-day-old 3rd instar larvae to determine the optimal concentration and repression time. When the concentration of *dsACP* was 2000 μg∙mL^−1^, the silencing efficiency was greatest at 61.5% (*p* < 0.05). There was no significant difference when the concentration of *dsACP* was 500 μg∙mL^−1^ (Figure 3A). The relative expression of the *HiACP* gene on different days following the *dsACP* injection was also analyzed. Silencing efficiency was best on the 3rd day (1.5 μL, 2000 μg·mL^−1^)) and reached 81.9% (*p* < 0.05) (Figure 3B).

The changes in fatty acid content in *dsACP*-injected 3rd instar larvae on the 3rd day after interference were studied using the RNAi method (Table 2). After interference, the total fatty acid content decreased by 13.13% (*p* < 0.05), and lauric acid (C12:0) decreased by 20.22% (*p* < 0.05). After interference with *dsACP*, the percentage of SFA in total fatty acid decreased by 2.44% (*p* < 0.05), while the percentage of UFA in total fatty acid increased by 4.94% (*p* < 0.05).

**Figure 4:**
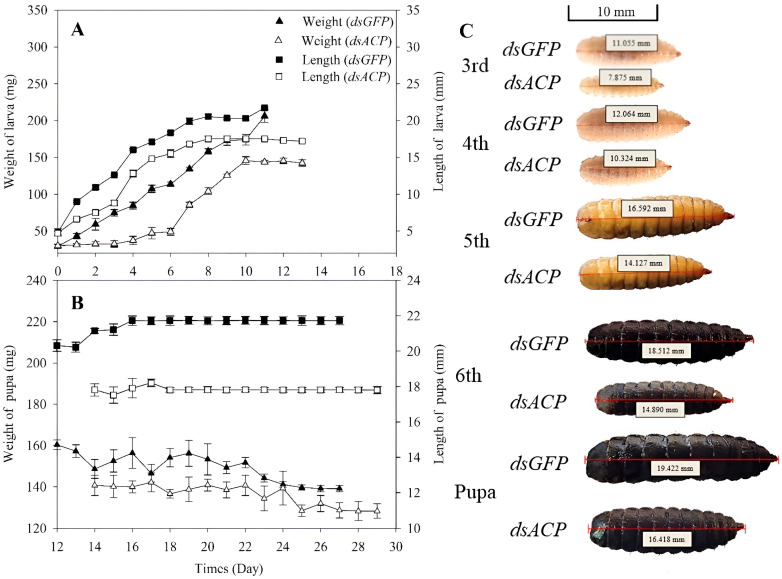
Changes in body weight, length, and appearance of larvae and pupae with time in one-day-old 3rd instar larvae of *H. illucen* after *dsACP* (2000 μg∙mL^−1^) injection of 1.5 μL. (**A**): Changes in body weight and length of larvae with time. (**B**): Changes in body weight and length of pupa with time. The 6th instar larva is also called larval in the pre-pupa stage, so it is also classified as a pupal stage. The left y-axis values are the mean of the average weight of larvae and the right y-axis indicates the length of *H. illucnens* larvae. (**C**): Appearance changes of larva and pupa of different instars. The photos of larva and pupa were collected at the 3rd day of the corresponding instar. Mean ± SE was calculated from three biological replicates, and each biological replicate contained 50 3rd instar larvae.

We studied the influence of *HiACP* interference on the growth of 3rd instar larvae of *H. illucens*. After injecting *dsACP*, the 3rd instar larvae were continuously observed for 30 d. The 3rd instar larvae injected with *dsACP* grew to 5th instar larvae. Compared to the control 5th instar larvae, the treated larvae had an average body weight that was 44.85 mg less and an average body length that was 3.09 mm shorter (Figure 4A). These values were significantly different from the control group (*p* < 0.05). When the 3rd instar larvae grew to pre-pupae, we observed that the weight of the average pre-pupa was 14.59 mg less, and the average pre-pupa length was 3.82 mm less than the control (Figure 4B). These reductions were significantly less than the control (*p* < 0.05).

**Figure 5:**
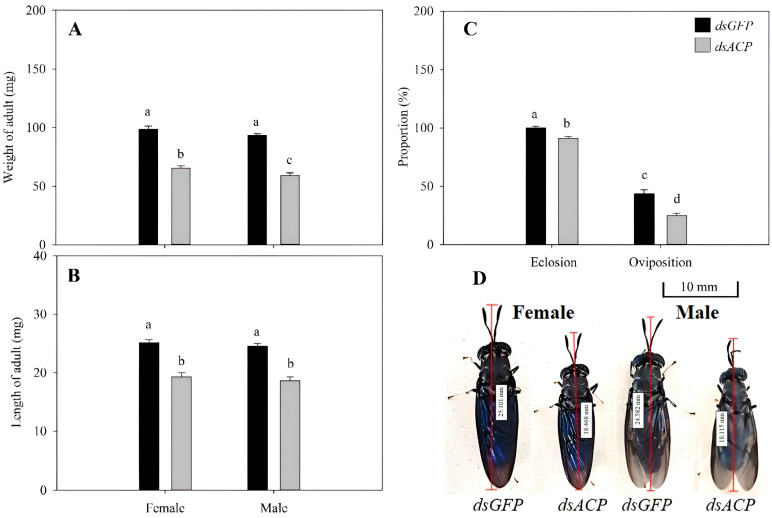
Changes in the body weight, length, eclosion, oviposition, and appearance in adults after the one-day-old 3rd instar larvae of *H. illucen* injected with 1.5 μL *dsACP* (2000 μg∙mL^−1^) grew to adults. (**A**): Changes in the weight of female and male adults. Y-axis values are the means of the average weight of adults. (**B**): Changes in the length of female and male adults. Y-axis values are the mean of the average length of adults. (**C**): Changes in the proportion of eclosion of adults and oviposition of female adults. Y-axis values are the means of the proportion of eclosion and oviposition. (**D**): Appearance changes of female and male adults. The pictures of adults were collected on the first day after molting. Every adult grew from a one-day-old 3rd instar larva of *H. illucen* injected with 1.5 μL *dsACP* (2000 μg∙mL^−1^). Mean ± SE was calculated from three biological replicates, and each biological replicate contained 50 one-day-old 3rd first instar larvae that grew to the adult stage. Bars marked with different letters indicate significant differences between treatment and control (Student’s *t*-test, *p* < 0.05).

After the 3rd instar larvae interference treatment, individuals grew to the adult stage, the adult male weight was reduced by 34.06 mg, and the adult female weight was reduced by 33.05 mg compared to the control. These changes were significantly different from the control group (*p* < 0.05) (Figure 5A). The experimental group’s adult male body length was reduced by 5.9 mm, and the adult female body length was reduced by 5.8 mm. These reductions were significantly different from the control group (*p* < 0.01) (Figure 5B). The adult eclosion rate was reduced by 8.86%, and adult female oviposition was reduced by 18.52% (Figure 5C). These values were significantly less than those in the control (*p* < 0.05).

**Figure 6:**
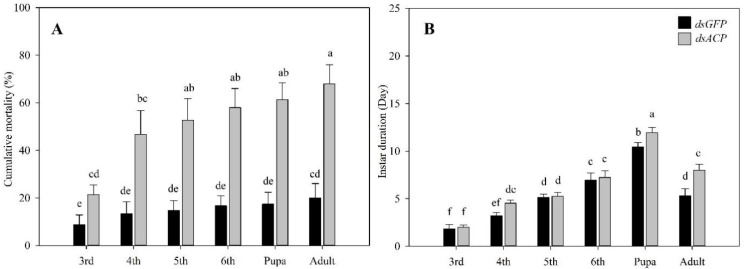
Cumulative mortality and instar duration of one-day-old 3rd instar larvae of *H. illucen* after *dsACP* (2000 μg∙mL^−1^) injection of 1.5 μL. (**A**): Cumulative mortality in different instars of 3rd instar larvae injected with *dsACP*. Y-axis values are the means of cumulative mortality in the different instars. (**B**): The duration of 3rd, 4th, 5th, 6th instar larvae, pupae, and adults. Y-axis values are the means of duration at different instars. Mean ± SE was calculated from three biological replicates, and each biological replicate contained 50 one-day-old 3rd instar larvae grown to different instars. Bars marked with different letters indicate significant differences between treatment and control (Student’s *t*-test, *p* < 0.05).

On day 40, after injecting *dsACP*, the treated 3rd instar larvae had developed into adults. The cumulative mortality of the experimental group was 68.00%, which was significantly greater than the control group (*p* < 0.01) (Figure 6A). We also found that after injecting *dsACP* into the 3rd larvae, there was a significant increase in instar duration. The duration of 4th instar larvae, pupae, and adults was significantly longer than the control group. The duration of 4th instar larvae increased by 1.3 d, pupal duration increased by 1.5 d, and the adult stage increased by 2.7 d. These increases were significantly different from the control group (*p* < 0.01) (Figure 6B).

## 4. Discussion

Fatty acid synthase plays a crucial role in the fatty acid metabolism of insects, and ACP is an important functional cofactor that directs the production of various fatty acids in insect tissues. We studied ACP deficient *H. illucens* to determine the potential function of this key FAS cofactor. Inhibition of ACP expression during the larva stage directly down-regulated the content of common fatty acids stored in the tissues. This effectively reduced the growth of *H. illucens*. The effects included a reduced survival rate, reduced body size and mass, increased development duration, and reduced female reproduction. These results indicated that ACP could have a great impact on fat storage and the growth of *H. illucens*. Advance editing of this gene might help improve fatty acid production and conversion efficiency of organic waste by *H. illucens*.

The development of *H. illucens* larvae can be affected by various extrinsic factors such as temperature, gut microbes, food nutrition, and xenobiotics [23,54,55]. Intrinsic factors, such as the expression level of metabolic genes, also perform vital roles during the developmental process. Our result exhibited that *HiACP* was expressed at the highest level during the egg stage of *H. illucens* (Figure 2A). During embryogenesis, a large amount of energy is needed to complete the transformation from egg to 1st instar larva. Lipids, consisting of glycerol and fatty acids, are the major fuel source during this development stage [56]. Similar to *H. illucens*, a large number of fatty acids occur in the oocytes of mosquitos. These have been termed maternal fatty acids [57]. Fatty acid deficiency can reduce the egg lifespan of insects and hence impair the fitness of eggs [57,58]. These results confirm that, in *H. illucens*, *HiACP* regulates the biosynthesis of intra-egg fatty acids and is involved in the development of the embryo to larval stages. After the egg hatched, the expression level of the *HiACP* gene increased during the *H. illucens* growth. This indicated a gradually increased storage of fatty acids in the gut and fat body of larvae (Figure 2B). This energy source could be used for pupation and subsequent adult eclosion. In adult female *Bombyx mori*, the *ACP* gene was highly expressed in the unfertilized egg, flight muscle, and fat body tissues. The adult *B. mori* midgut had a relatively lower expression level than those found in other tissues [59], which is inconsistent with our results. This could be caused by the degenerated gut tract of the silkworm, which does not participate in food digestion and fatty acid metabolism.

In mammals, fatty acid synthase mainly produces C16 fatty acids, whereas insect fatty acid products are evenly distributed in carbon chain lengths, reaching C18 [60]. Large amounts of C18 fatty acids, such as stearic acid (C18:0), oleic acid (C18:1), linoleic acid (C18:2), and linolenic acid (C18:3), have been identified in many insects, including crickets, grasshoppers, mealworms, and houseflies [61,62,63]. *H. illucens* larvae contain a large amount of lauric acid (C12:0), palmitic acid (C16:0), and oleic acid. Our results demonstrated that SFAs are dominant in the *H. illucens* larva, which is consistent with previous studies of this species [33,64]. Lauric acid (C12:0) was the most abundant fatty acid in *H. illucens* larvae, a result that differs from previous data. *H. illucens* contains high levels of lauric acid and serves as a useful SFA supplier for the food, cosmetics, and poultry industries [65]. Using the *H. illucens* RNAi system, we found that lauric acid content decreased by 20.22% in the 3rd instar larva after silencing of *HiACP*. However, other C16 and C18 SFA and MUFA compounds were also simultaneously down-regulated. No fatty acid was observed to decrease significantly compared with its other analogs. This result implies that *HiACP* mainly participates in the elongation step of fatty acid biosynthesis in *H. illucens* and might not be involved in the decoration and termination reaction of the hydrocarbon chain. Functional genes that contribute to the composition of specific fatty acid types (e.g., lauric acid) may be uncovered by future RNAi studies.

RNAi treatment had significant effects on the growth and development of 3rd instar *H. illucens* larvae. Inhibition of *HiACP* decreased fatty acid storage in tissues and impaired triglyceride synthesis in RNAi-treated individuals. When the energy storage ability of insects was compromised, their body size, duration of development, and fertility were also affected. This result indicated that *HiACP* is essential for the nutrient digestibility and energy storage of *H. illucens*. ACPs have exhibited similar functions in other animals. For example, the downregulation of genes related to fatty acid synthesis can induce dysfunctions in adipose tissue and lead to fat loss and weight loss in rats [66]. Similarly, RNAi treatment of the *FAS* gene disturbed the rate of midgut digestion and caused reduced lipid synthesis in *Aedes aegypti* [67]. The fatty acid synthesis pathway is a basic biological process, the products of which are widely involved in the physiology of the immune and other cell signaling systems [68]. For example, lauric acid has bactericidal activity [69], so a decrease in lauric acid content triggered by ACP interference, as observed here, might suppress the immune function of *H. illucens* against bacterial pathogens. The chain extension reaction of the fatty acid synthesis process is also used by the insect hormone biosynthesis process [70,71,72], which indicates that the ACP downregulated induced disturbance of the fatty acid elongation reaction might diminish the hormone homeostasis of *H. illucens.* We assume that this hormone dysfunction might have a wider influence on insect physiology and ecology.

RNAi treatment of *H. illucens* demonstrated that *HiACP* is a core gene involved in the fatty acid synthesis process. This gene cannot precisely govern the production of specific fatty acid content and the composition of fatty acids stored in *H. illucens* tissues. It appears to act as a “general controller” of the fatty acids that regulate fatty acid composition and storage in *H. illucens*. This knowledge could be used for expanding the fatty acids produced by the *H. illucens* rearing industry.

## 5. Conclusions

We demonstrated that the *HiACP* gene regulates the biosynthesis of fatty acids. This gene affects the growth of *H. illucen* larvae, adult eclosion rate, and adult oviposition. These effects helped reveal the molecular mechanism and function of the *HiACP* gene and the process of fatty acid regulation. Future research should focus on the optimization of the fatty acid regulatory mechanisms and the expression of high-quality fatty acids in vitro.

## Figures and Tables

**Table 1:** Sequences of primers for DNA cloning, dsRNA synthesis, and qRT-PCR.

Primer	Forward Primer	Reverse Primer	Product Length (bp)	Purpose
*HiACP*	AAAAATGTCGCTGTCACAGGGT	CAACAAAACGATGAACCCGC	501	cDNA
*dsACP*	T7-CGCTGTTTATTGAGCCGTGC	T7-TTCTCGGCATCTGAATCGGG	395	RNAi
*dsGFP*	T7-AGATCCGCCACAACATCGAG	T7-GTCCATGCCGAGAGTGATCC	204	
*qHiACP*	GATGCTCCATCGACAATGCG	GGACGCGTTCACGAATTTCT	137	qRT-PCR
*β-actin*	AGGAGACGAAGCACAAAGCA	AGTCCAAAGCGACGTAGCAG	150	

T7: GATCACTAATACGACTCACTATAGGG.

**Table 2:** Fatty acid composition (g/100 g) of 3rd instar larvae after *dsACP* injection.

Fatty Acid	*dsGFP*	*dsACP*
C10:0	0.21 ± 0.00	0.17 ± 0.01 *
C12:0	9.94 ± 0.08	7.93 ± 0.06 **
C14:0	2.31 ± 0.02	1.91 ± 0.01 **
C15:0	0.11 ± 0.01	0.06 ± 0.02
C16:0	5.24 ± 0.23	4.94 ± 0.23
C16:1	1.03 ± 0.04	0.82 ± 0.01 **
C18:0	0.94 ± 0.06	0.88 ± 0.03
C18:1	5.09 ± 0.04	4.6 ± 0.14 *
C18:2	2.84 ± 0.18	2.76 ± 0.28
C18:3	0.31 ± 0.03	0.27 ± 0.05
SFA	18.75 ± 0.15	15.89 ± 0.25 **
MUFA	6.12 ± 0.07	5.42 ± 0.12 **
PUFA	3.15 ± 0.21	3.03 ± 0.32
Total FA	28.02 ± 0.17	24.34 ± 0.24 **

Mean ± SE was calculated from three biological replicates and four technology replicates; each biological replicate contained 15 one-day-old 3rd instar larvae. The larvae injected 1.5 μL (2000 μg·mL^−1^) *dsACP* were collected on the 3rd day. *: 0.01 < *p* < 0.05, **: *p* < 0.01.

## Data Availability

Data will be available upon reasonable request to authors.
